# Team approach to polypharmacy evaluation and reduction: feasibility randomized trial of a structured clinical pathway to reduce polypharmacy

**DOI:** 10.1186/s40814-023-01315-0

**Published:** 2023-05-18

**Authors:** Dee Mangin, Larkin Lamarche, Gina Agarwal, Abbas Ali, Alan Cassels, Kiska Colwill, Lisa Dolovich, Naomi Dore Brown, Barbara Farrell, Karla Freeman, Kristina Frizzle, Scott R. Garrison, James Gillett, Anne Holbrook, Jane Jurcic-Vrataric, James McCormack, Jenna Parascandalo, Julie Richardson, Cathy Risdon, Diana Sherifali, Henry Siu, Sayem Borhan, Jeffery A. Templeton, Lehana Thabane, Johanna Trimble

**Affiliations:** 1 Department of Family Medicine, David Braley Health Sciences Centre, McMaster University, 100 Main Street West, 5th Floor, Hamilton, ON L8P 1H6 Canada; 2 Dept. of General Practice, University of Otago, Christchurch, New Zealand; 3 University of Victoria, 3800 Finnerty Rd, Victoria, BC Canada; 4 University of Toronto, 144 College Street, Toronto, ON Canada; 5 Bruyère Research Institute, 43 Bruyère Street, Ottawa, ON Canada; 6 University of Alberta, 6-60 University Terrace, Edmonton, AB Canada; 7 University of British Columbia, 2405 Wesbrook Mall, Vancouver, BC Canada

**Keywords:** Polypharmacy, Deprescribing, Multi-morbidity, Patient safety, Primary care

## Abstract

**Background:**

Polypharmacy is associated with poorer health outcomes in older adults. Other than the associated multimorbidity, factors contributing to this association could include medication adverse effects and interactions, difficulties in managing complicated medication regimes, and reduced medication adherence. It is unknown how reversible these negative associations may be if polypharmacy is reduced. The purpose of this study was to determine the feasibility of implementing an operationalized clinical pathway aimed to reduce polypharmacy in primary care and to pilot measurement tools suitable for assessing change in health outcomes in a larger randomized controlled trial (RCT).

**Methods:**

We randomized consenting patients ≥ 70 years old on ≥ 5 long-term medications into intervention or control groups. We collected baseline demographic information and research outcome measures at baseline and 6 months. We assessed four categories of feasibility outcomes: process, resource, management, and scientific. The intervention group received TAPER (team approach to polypharmacy evaluation and reduction), a clinical pathway for reducing polypharmacy using “pause and monitor” drug holiday approach. TAPER integrates patients’ goals, priorities, and preferences with an evidence-based “machine screen” to identify potentially problematic medications and support a tapering and monitoring process, all supported by a web-based system, TaperMD. Patients met with a clinical pharmacist and then with their family physician to finalize a plan for optimization of medications using TaperMD. The control group received usual care and were offered TAPER after follow-up at 6 months.

**Results:**

All 9 criteria for feasibility were met across the 4 feasibility outcome domains. Of 85 patients screened for eligibility, 39 eligible patients were recruited and randomized; two were excluded post hoc for not meeting the age requirement. Withdrawals (2) and losses to follow-up (3) were small and evenly distributed between arms. Areas for intervention and research process improvement were identified. In general, outcome measures performed well and appeared suitable for assessing change in a larger RCT.

**Conclusions:**

Results from this feasibility study indicate that TAPER as a clinical pathway is feasible to implement in a primary care team setting and in an RCT research framework. Outcome trends suggest effectiveness. A large-scale RCT will be conducted to investigate the effectiveness of TAPER on reducing polypharmacy and improving health outcomes.

**Trial registration:**

clinicaltrials.gov NCT02562352, Registered September 29, 2015.

**Supplementary Information:**

The online version contains supplementary material available at 10.1186/s40814-023-01315-0.

## Key messages regarding feasibility

1. What uncertainties existed regarding the feasibility?
  The extent to which the implementation of TAPER was possible in a primary care setting for clinicians, participants, and researchers
  The extent to which there appeared to be any evidence of a possible effect TAPER had on a range of outcome measures compared to usual care

2) What are the key feasibility findings?
  TAPER is feasible to implement in a routine clinical practice setting in primary care.
  A sufficiently high proportion of participants and clinicians were willing to engage in the intervention.
  The direction of the effect on outcomes appears to favor TAPER versus usual care.

3) What are the implications of the feasibility findings for the design of the main study?
  Challenges uncovered were those mitigated by adaptations in process and would not prevent the success of a larger randomized controlled trial.
  There were trends in outcomes that suggested an effect is worthwhile testing in a large adequately powered randomized controlled trial.

## Background

Polypharmacy is commonly defined as taking five or more long-term medications [1, 2]. Canadian older adults living in the community taking five or more medications have nearly doubled between the years of 1998–2008 from 13 to 27–30% [3–5]. Polypharmacy is associated with negative health outcomes in older adults, with increased risk of mobility-related functional decline, falls, hospitalizations, impaired cognition, and reduced quality of life [6–10]. Although polypharmacy is a reality of multimorbidity and drugs are beneficial for the management of symptoms in older adults, polypharmacy also contributes to the burden of treatment, and the balance of benefits and harms can change with time and aging [11]. This treatment burden may contribute to the association of polypharmacy with negative health outcomes. Medication adverse effects, medication interactions, complex regiments, and reduced medication adherence are factors that independently or in combination contribute to treatment burden and may also compromise the patient’s ability to cope [8, 10, 12–15]. For example, there is an increased risk of adverse drug reactions (ADRs) as the number of medications increases (2 medications have a 13% risk, 5 medications have a 58% risk, and 7 or more have a 82%) [12, 16]. According to a Canadian study, many ADRs are preventable [17].

There is increasing interest in processes to reduce polypharmacy; this involves reviewing a patient’s medications with the purpose of reducing the number and/or dose of medications, as well as the goal of reducing the harms and/or burden of polypharmacy [18]. These processes have generally been categorized as explicit (criteria-based tools) and implicit (judgement-based tools) [19]. Several explicit tools are available to guide clinicians when making decisions about deprescribing and/or identifying inappropriate medications (the Beers list, STOPP) [20, 21]. Numerous studies of various designs (retrospective, prospective, cohort, and randomized controlled trials) have shown such tools can predict a significant proportion of hospitalizations due to the adverse drug reactions [22–26], including in a primary care setting [27, 28]. To support a more individual focus in reducing particular drugs or classes, medication-specific deprescribing guidelines are available [29–33] such as those developed by the Bruyère Research Institute in Ottawa, Canada [34].

Despite the benefits of these types of approaches, many tools such as Beers and STOPP are only designed to flag those medications associated most commonly with drug-related problems in older adults. It is possible for a patient with polypharmacy to be taking no medications on these lists, be treated according to guideline-congruent care appropriate for single disease management, and yet experience multiple adverse effects related to their drug treatment [35, 36]. Furthermore, some tools do not constitute a patient-centered or patient-focused approach to care; they do not consider patient preferences. By including the patient’s voice (goals, priorities, and preferences for treatment) in situations where polypharmacy includes multiple medications that all may offer potential benefits, it is possible to help prioritize a medication list to reduce polypharmacy.

Most efforts undertaken to address polypharmacy have not explicitly considered patient preferences or priorities. We identified one approach, Systematic Tool to Reduce Inappropriate Prescribing (STRIP) [37], which identifies potentially inappropriate prescribing with the consideration of patient preferences. Inappropriate prescribing can arise when the risks of using a medication regiment outweigh the benefits [38]. For example, inappropriate prescribing can involve failing to use a safer alternative, omitting use of a beneficial or appropriate treatment, using an incorrect treatment regime such as dosage, or when a drug has significant interactions with another drug or patient’s comorbidities. A large randomized controlled trial is currently underway in Europe (OPERAM, clinicaltrials.gov) that aims to evaluate the implementation and effectiveness of STRIP within a hospital setting.

TAPER, team approach to polypharmacy evaluation and reduction, operationalizes a clinical pathway aimed to reduce polypharmacy. The theoretical basis for TAPER has been described elsewhere, but in summary, it is designed to address known barriers to deprescribing at the patient, provider, and system level as well as mapping to established models of care [39]. Like STRIP, this approach also considers patient preferences and priorities. However, TAPER has explicit consideration of priorities and preferences explicitly related to medications and individual medication experience and was developed for use within usual primary care setting workflow, with potential for adaptation to other contexts. Briefly, it is a model for addressing polypharmacy that involves the team of patient, pharmacist, and physician, who all bring particular expertise. TAPER uses sequentially linked consultations with both a pharmacist and physician. These consultations draw together the patient’s expertise and the effects of their medications on them, the clinicians’ expertise in medications, the context of their clinical state and circumstances (including multimorbidity), and their longitudinal relationship with the patient. TAPER is grounded in the idea of a “drug holiday” — the pathway is framed as a longitudinal structured “pause and monitor” process, with a patient-focused approach. Evidence-informed tools support this process, flagging potentially inappropriate medications as a “machine screen” and providing guidance around tapering and monitoring, and there are evidence summaries on risks and benefits in older adults. An underpinning secure digital platform (TaperMD) integrates these elements in a shared electronic record platform accessible by the pharmacist and primary care physician that also allows incorporation into clinicians’ existing individual record systems. The primary purpose of this study was to determine the feasibility of implementing TAPER in a primary care setting in patients 70 years of age or older and who are on five or more long-term medications, and the secondary purpose was to perform initial hypothesis testing.

### Research questions and hypotheses

Nine research questions for this study are outlined in Table 1. The categorization of the feasibility sub-questions as process, resources, management and scientific in Table 1 are based on Thabane, Ma, Chu, Cheng, Ismaila, and Rios [40].

**Table 1:** Feasibility question, outcome, method of collection, pre-specified criteria for success

Category	Feasibility research question	Outcome (method of collection)	Pre-specified criteria for success
Process	What is the recruitment, refusal, and drop-out rate of participants, pharmacists, and family physicians?	Number of clinicians and participants enrolled Number of patient participants enrolled, not interested, and dropped out of study before 6-month period (master file)	50% of invited pharmacists and family physicians enrol 20% of invited patients enrol Less than 20% enrolled patients withdraw
	What are the challenges with determining to extent to which participants meet eligibility criteria?	Description (recruitment file, researcher call notes)	Ability to mitigate challenges that arise during recruitment and eligibility checking in larger trial
	What is the nature of challenges in terms of participants’ understanding of, and ability to respond to, the surveys?	Description (field notes by research team)	Ability to mitigate challenges that arise during data collection appointments in larger trial
	What is the number and nature of any instances of unblinding?	Description (field notes by the research team)	Less than 10 instances of unblinding; all instances can be mitigated in larger trial
Resources	What is the length of time to complete the surveys?	Duration of visits, number of participants needing multiple visits to complete collection for time point (program records)	Less than 50% of participants require more than 1 visit to complete data collection; less than 2 h required for data collection visits
	How much travel time does the research team do to complete visits?	Estimated time (recruitment file)	Less than 50% of visits require ≥ 30 min of travel time
Management	What is the nature of data entry problems?	Description (field notes by research team)	Ability to mitigate common data entry errors (e.g., data missing at random, data syncing issues, problems with database) in larger trial
Scientific	What is the nature of any adverse events associated with the intervention or study process?	Number and description (field notes by research team, 1 week, 3- and 6-month check-in data collection forms)	No serious adverse events associated with the intervention or study process
	What is the variance, potential floor and ceiling effects, for research outcomes?	Range of scores for each variable (according to survey)	No floor or ceiling effects detected; moderate to high levels of variance detected

It was hypothesized that process, resources, management and scientific indicators of feasibility will be demonstrated, with the identification of implementation challenges which could be mitigated in the design of a larger randomized controlled trial.

## Methods

### Study design and setting

We conducted a prospective 1:1 single-blinded randomized controlled feasibility trial. At the end of the study, the control group was offered the intervention. The findings of this trial will help in conducting a larger scale randomized controlled trial (clinicaltrials.gov, no. NCT02942927). This study’s outcome measures of interest for hypothesis testing are registered at clinicaltrials.gov (no. NCT02562352). The study was carried out in Hamilton, Ontario, by the McMaster University, Department of Family Medicine at the McMaster Family Health Team (MFHT). Family Health Teams are primary care organizations that formally link physicians and a variety of healthcare professionals together [41]. Patients who were 70 years or older, rostered with physicians who are part of the McMaster University Sentinel and Information Collaboration Practice-Based Research Network (MUSIC), and who were taking 5 or more long-term medications were eligible to participate in the study.

### Participants and participant recruitment

All 31 family physicians and 3 clinical pharmacists at MFHT were invited to participate. Patient participants were recruited from the McMaster University Sentinel and Information Collaboration Practice-Based Research Network (MUSIC) network through the already established Health TAPESTRY program (Health Teams Advancing Patient Experience: Strengthening Quality) [42, 43]. Participants were eligible if they were 70 years of age or older and taking five or more long-term medications at the time of their initial assessment. Participants had to be willing to try medication discontinuation. Participants were excluded if they had a recent comprehensive medication review (within 6 months), had inadequate English or cognitive skills to understand and respond to the surveys, or had a terminal illness or other circumstances that would preclude them from a 13-month study period. We aimed to recruit 30–40 participants as we felt this was adequate to test the processes required for a larger RCT and collect adequate data to access the proposed tools.

### Allocation and randomization

Participants were randomly allocated to either the intervention or control group (1:1 ratio) using variable block sizes of 2, 4, 6, or 8 through REDCap (Research Electronic Data Capture) [2, 44], a secure web-based software that can be used for both randomization and data collection and management. The randomization sequence was generated and maintained by the Biostatistics Unit at the St. Joseph’s Healthcare Hamilton.

### Blinding process

Participants were not blinded to their group allocation. Participant blinding in this study was neither necessary nor practical as the focus is feasibility of the effectiveness of reducing medications rather than a trial of the pharmacological effect of a drug. The family physicians and pharmacists were masked to allocation as they were not aware of whether the appointment they were completing was for an intervention or 6-month waitlist control participant. Procedures were executed to ensure that the participants did not accidently unblind the researcher completing the outcome assessments. The effectiveness of the blinding process was evaluated at the completion of the study.

### Procedures

To boost potential enrollment, participants who were eligible were contacted by their family doctor (and not the research team directly) by mail with an invitation letter outlining the study. Participants returned a prepaid postage envelope to the study team indicating their interest in the study. Those who expressed their interest were then contacted by the study team to be screened for eligibility and to formally go through the consent process. After consent, a research data collection session was booked according to group allocation. The session was anticipated to take 1–2 h and was done either at the patients’ home or at the research facility. Outcome measures were collected by a researcher at baseline and then again after 6 months. Follow-up symptom assessments were also conducted at 1 week, 3 months, and 6 months by a researcher over the phone. The control group received the usual standard of care. After all data was collected at 6 months, control participants were offered the intervention. Baseline recruitment began in November 2016 and ended in December 2017. Six-month follow-up collection started in May 2017 and ended in June 2018.

### Intervention

**Fig. 1:**
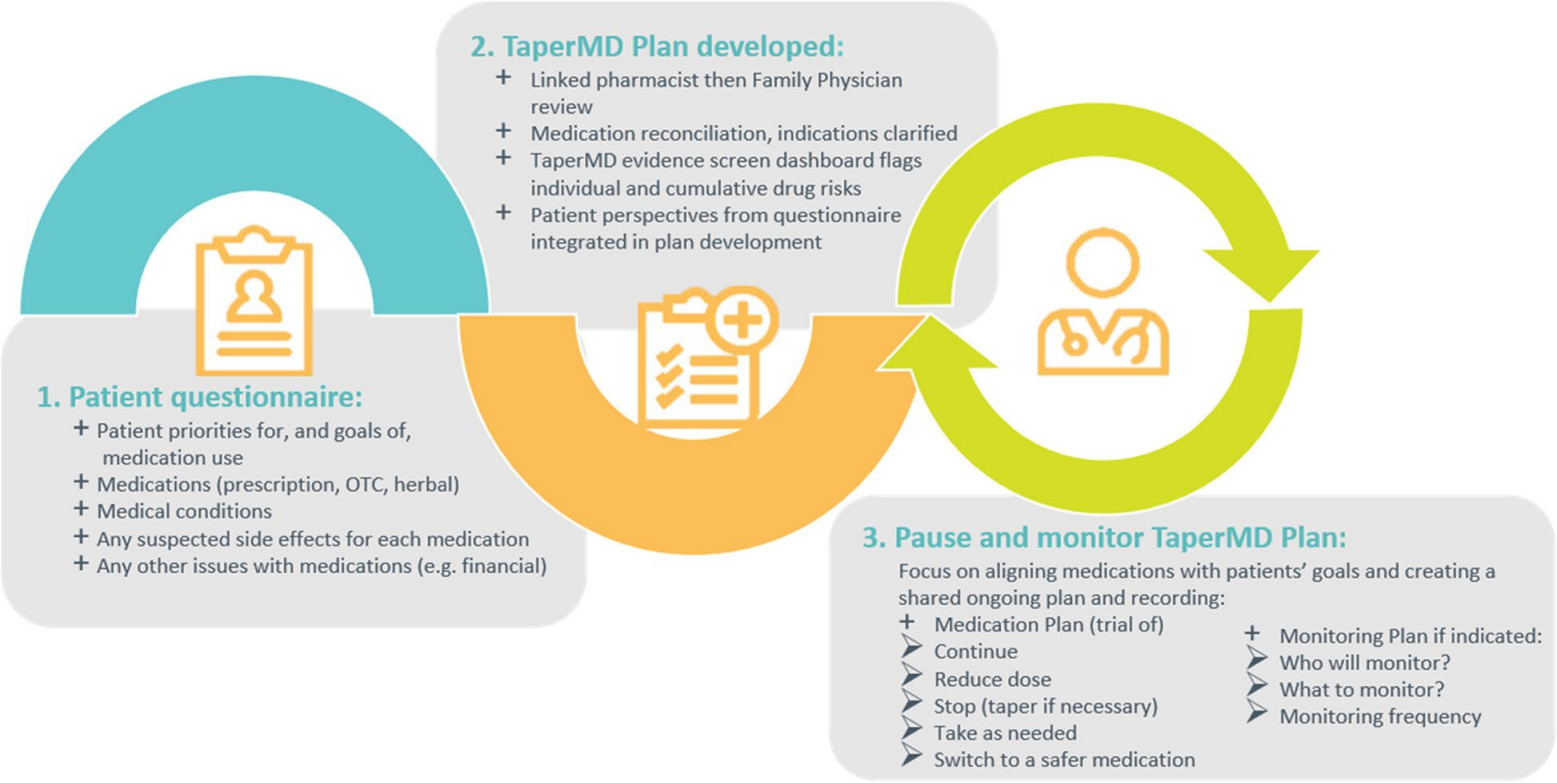
Structured medication discontinuation clinical pathway

The intervention operationalized a clinical pathway (TAPER) aimed to reduce polypharmacy. It involved a cooperative team-based structure for a complete medication review by the pharmacist and the physician aimed at reducing medication burden. The approach collected foremost patient’s priorities, preferences, and experience of their medications. It then used explicit evidence and tools to automatically screen for and flag potential inappropriate medications or combinations through use of an integrated e-tool “machine screen” within the pathway. The objective was to combine this range of existing evidence, any available specialized tools, provide evidence for discontinuation management, and integrate this with the patient’s preferences to develop a collaborative, longitudinal plan as a “pause and monitor” trial of medication discontinuation. Information was entered, stored, and shared via an online platform, TaperMD, which provided a shared platform for recording and teamwork between pharmacist and physician. All clinicians were provided with training on TaperMD with an initial overview tutorial of around 30 min and then a “ride-along” with a researcher specifically available at the consultation time during use with the first patient to answer any questions. The pharmacists were provided with additional 1-h, in-person training on TaperMD. Video tutorials on each section of the tool were also available on an internal YouTube channel. A summary of the pathway is found in Fig. 1, following a description of each step below.

#### Step 1: collection of participant information at baseline

The research assistant interviewed the participant about their goals, preferences, and priorities, recorded medication side effect risk factors, perceived medication side effects, and entered an initial current list of medications using the dispensing pharmacy list, information in the electronic medical record, and the participant into TaperMD.

#### Step 2: clinical pharmacist consultation

The clinical pharmacist completed a comprehensive review of the participant’s medications (gathered by the research assistant from the patient) and medical conditions (from the electronic medical record system or from the patient themselves) in an appointment with the participant. The pharmacist reviewed medications that were entered by the research assistant in TaperMD, along with the medications on the patients’ dispensed list, and created a reconciled, current medication list. TaperMD automatically created the “machine screen” which flags potential inappropriate medications, which could be suitable for discontinuation or dose reduction. These tools and lists highlight potentially inappropriate medicines (and reasons) in older adults in a simultaneous multidrug view consistent with a multimorbidity approach. Specific medication dimensions flagged include standard interaction checking, potentially inappropriate medicines in older adults drawn from assessment of a wide range of jurisdiction specific lists [19], drugs contributing to anticholinergic burden score, QT prolonging drug burden, hypotensive drug burden, serotonergic drug burde,n and deprescribing guidelines and algorithms where these are available. The screens included are described in detail in Additional file 5. Informed by this information, clinical judgement and knowledge and the patient’s priorities, a list of suitable recommendations for appropriate discontinuation, were made. An initial plan was developed and stored in TaperMD for family physician review.

#### Step 3: family physician consultation

Within approximately 1–2 weeks following the pharmacist consultation, the family physician met with the participant for an extended appointment. The family physician reviewed the pharmacist recommendations and patient priorities and the reports of medication effects prior to the appointment. Using the same principles, and the pharmacist’s suggestions, at the consultation with the patient, a discussion of the final assessment of the medications suitable for a trial of “pause and monitor” occurs, and a plan was made. The physician validated or adjusted recommendations within the pharmacist’s initial plan. The final plan includes information about the following: *what* will be discontinued, *what* will be monitored, *who* will monitor (patient pharmacist or physician), *how often* and *when*, and *when* would medication restarting be considered.

#### Clinical monitoring

Participants attended monitoring visits after the initial plan, as clinically indicated by the particular drugs selected, during the “pause and monitor” phase. Planned monitoring was recorded in TaperMD.

### Data collection

Demographic information (collected at baseline) and research outcomes (listed below and collected at baseline and 6 months) were collected by the research assistant. Participant characteristics (age, gender, and income) were self-reported. The Charlson Comorbidity Index [45] was used to assess burden associated with chronic conditions via chart audit [46]. Medication-related information (beliefs about medications [47], current medication list with indication for prescribing [48] (was also collected via patient self-report, chart audit, and using pharmacy data.

### Feasibility outcomes

We considered four categories of feasibility questions to determine whether a larger randomized trial would be feasible [39]. Specifically, process outcomes were included to assess the feasibility of the steps that need to take place for a successful main trial, resource outcomes were collected to assess time and budget challenges for the main trial, management outcomes to determine challenges of human or data management, and scientific outcomes were considered to assess intervention safety and outcome variance. Each feasibility research question, feasibility outcome, the method of collection, and pre-specified threshold for success have been outlined (Table 1).

### Proposed outcome measures for process, performance, and hypothesis testing

#### Primary outcome measures

The primary outcomes were the number of prescribed and prescribable medications, at 6 months after baseline. For intervention patients, this information was collected 6 months after the patient met with the physician (step 3), and for control patients, it was collected 6 months after the baseline study visit appointment with the researcher (step 1). The medications were gathered from the medication list in TaperMD, which was the reconciled medication list done by the study pharmacist at baseline and at 6 months and recorded changes made to medications during the intervention period. Medications were categorized as either prescribed, prescribable, or non-prescribable. Prescribed medications include all schedule 1 medications (medications requiring a prescription) [49] and any medications that were dispensed through a prescription or where a prescription was found in the electronic medical record (EMR). Prescribable medications are defined as schedule 2, 3, or unscheduled [49] medications that a medical physician or nurse practitioner could reasonably prescribe (e.g., vitamin D, vitamin B12, calcium carbonate, acetaminophen, ibuprofen) but are purchased over the counter rather than dispensed through a prescription.

#### Secondary outcome measures

Secondary outcomes included number of non-prescribable medications (defined as schedule 3 or unscheduled [49] medications that would not be prescribed by physicians or nurse practitioners but are purchased over the counter at the discretion of the patient, e.g., naturopathic preparations, homeopathic preparations, multivitamins), medication dose changes, quality of life, psychological distress, cognition, fatigue, nutritional status, physical functional capacity, falls, adverse events, healthcare utilization, and patient enablement. All were collected at baseline and at 6 months (Table 2). All outcomes were assessed using validated measures or custom self-report forms, or data was extracted from the EMR. Note, we also assessed the utility of the Flinders Fatigue Scale (to assess fatigue) in a few participants [50].

**Table 2:** Secondary outcome measures for assessment of process and performance

Outcome	Measure or source	Details of measure	Scoring
Quality of life	Short-Form Health Survey (SF36-V1)	• Eight domains: role-physical, bodily pain, general health, vitality, social functioning, role-emotional, and mental health	0 to 100, with higher scores = higher quality of life
	EQ5D-5L [51, 52]	• Five domains with 5-point scales: mobility, self-care, usual activities, pain/discomfort, and anxiety/depression • Overall health rated on scale from 0–100 (“worst imaginable health” to “best imaginable health”)	The Canadian Index was used: -0.148 to 0.949, with higher scores = high quality of life
	World Health Organization Disability Assessment Schedule 2.0 (WHODAS) [53]	• Thirty-six items to assess health and disability across all diseases and is useful for multimorbidity • Six domains of functioning rated as 0–4 (none, mild, moderate, severe, extreme): including cognition, mobility, self-care, getting along, life activities, and participation	Summed the first 36 items 0 to 144, with higher scores representing higher disability
Psychological distress	Kessler Psychological Distress Scale [54]	• Ten items that produce a global measure of distress based on questions about anxiety and depressive symptoms experienced in last 4 weeks • 5-point scale (all of the time, most of the time, some of the time, a little of the time, and none of the time)	Did not include the question “during the last 30 days, how often did you feel worthless?” Scores for 9 items range 9 to 45, with higher values representing higher psychological distress
Cognition	Mini-mental status examination (MMSE) [55, 56]	• Eleven items for 5 domains of cognitive function: orientation, registration, attention, calculation, recall, and language	Overall score ranges 0 to 30 Severe impairment = 0 to 9 Moderate impairment = 10 to 19 Mild impairment = 20–25 Potentially normal = 26–30 [57]
Mobility-related fatigue	Avlund Mobility-Tiredness Scale [58]	• Indicated whether participant experiences fatigue performing 6 activities (yes = 1 or no = 0)	0 to 6, with higher scores representing greater fatigue
Mobility	MANTY (64)	• Three items: level of difficulty walking 2 km, walking 0.5 km, and climbing 1 flight of stairs • Participants indicate the following: manage without great difficulty, some difficulty, a great deal of difficulty, with the help of another person or unable to manage even with help	Descriptive
Nutritional status	Mini Nutritional Assessment Short-Form [59]	• Six items: identifies elderly patients who are malnourished or at risk of being malnourished • Has been used to measure decline in nutritional status due to polypharmacy (63)	0 to 14, with higher scores indicating a better nutritional status
Sleep	Pittsburgh Sleep Quality Index (65)	• Assesses 1-month period • Nineteen items grouped into 7 components which are weighted equally on a scale of 0–3	0 to 21, with higher scores indicating poorer sleep quality
Patient enablement	Patient Enablement Instrument (66)	• Six items about patient understanding of and coping with health issues as a result of a consultation with a healthcare provider • We changed the stem, “As a result of your visit to the doctor today” to “After a usual visit with your family doctor”	0 to 12, with higher scores indicating a stronger patient enablement
Grip strength	With a JAMAR hand dynamometer	• Done with a supported forearm (67) but alternating between right and left hands to prevent fatigue • Three trials per hand	Average of three trials Measured in kilograms
Number of falls	Electronic Medical Record	• The number of falls and those requiring medical attention • The number of health care providers seen by the patient • The number of visits to the providers related to each fall incident	Descriptive
Healthcare utilization	Electronic medical record Custom self-report form	• Number of hospitalizations, emergency room visits, or urgent care visits • Number of long-term care admissions • Number of family doctor or specialist visits • Medication use • Home care services • Professional care services • Support equipment use • Caregiver support • Home care visits	Descriptive

During each study visit, the researcher recorded any potential serious adverse events. We used a separate researcher as unblinding was possible. If present, a local serious adverse event form was completed and reported to the family physician. Potential side effects that were unmasked as result of the study were also collected from patient and clinical reports. Minor and serious adverse events were categorized using the FDA criteria. We did not collect or report on when medications were tapered or the order of tapering in this study, although the information was available in the patient’s EMR and TaperMD profile.

#### One week and 3- and 6-month follow-ups

Follow-up was done by telephone at 1 week and 3 and 6 months by a research assistant to capture any changes in side effects or symptoms (either positive or negative) and to record healthcare visits. In an open-ended question, patients were asked if they have experienced any worsening of, or improvement in, any side effects or symptoms. They were also asked how many times they visited their family physician or the emergency room since the last study appointment. This researcher was different to the researcher recording outcome data to maintain blinding as it was possible that unblinding could occur in symptom description. Serious adverse events were also extrapolated from these conversations; a detailed assessment of any adverse event was also recorded, and addressed by the Principal Investigator, and the patient’s usual primary care clinical setting.

### Sample size

The sample size for this study was based primarily on feasibility considerations [40, 60]. We aimed to recruit *n* = 36 participants (*n* = 18 per group). This is in line with general guidance for sample size for pilot trials aimed at assessing feasibility [53, 54].

### Statistical analysis

Results from this trial are reported in accordance with the CONSORT statements for pilot and feasibility studies (Additional file 2, [60]) and TIDieR checklist (Additional File 3, [55]). Baseline participant characteristics were reported in terms of mean (standard deviation) or median (first quartile, third quartile), depending on the distribution, for continuous variables and count (percentage) for categorical variables. We used descriptive statistics such as, count (percentage) to analyze the feasibility outcomes. The primary analyses were performed using intention-to-treat approach. Multiple imputation method was used to impute the missing data. In total, 5 datasets were generated and the pooled estimates were reported.

The primary outcomes, the number of medications (prescribed and prescribable) and number of prescribed medications, were analyzed using the Poisson regression with the treatment group as the covariate. The rate ratios (RRs) along with 95% confidence intervals were reported. The secondary continuous outcomes (shown in Table 2) were analyzed using the linear regression with treatment group as the covariate. The mean differences (MDs) along with 95% confidence interval were reported. All statistical tests were two sided at the level of significance 0.05. The data were analyzed using statistical software R version 3.5.1 [56]. Sensitivity analyses were performed to assess the robustness of the results of the primary analyses using per-protocol approach.

## Results

**Fig. 2:**
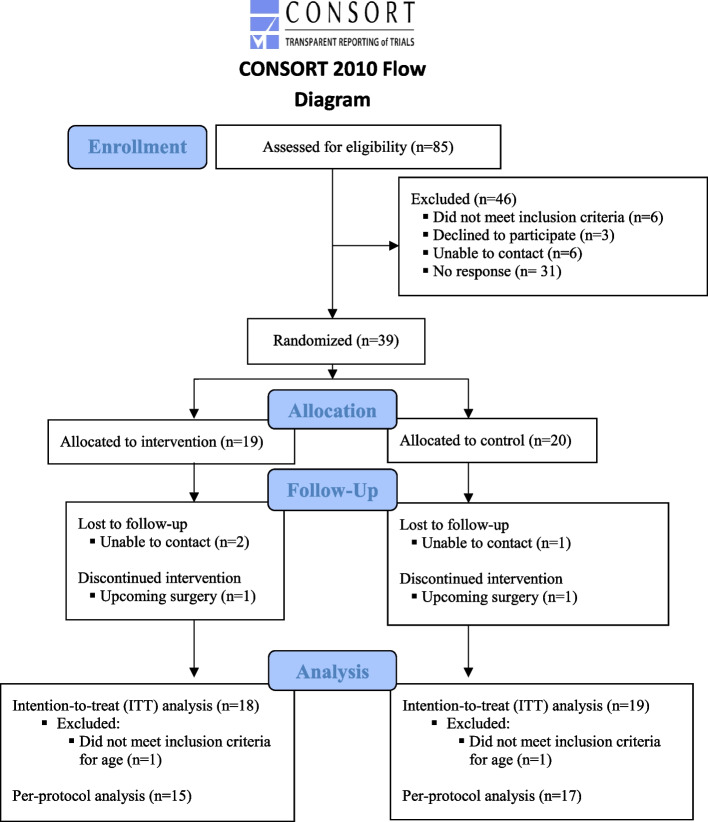
CONSORT participant flow diagram

Participant characteristics by group are reported in Table 3, and the results of the feasibility outcomes are presented in Tables 4, 5, 6 and 7, and the flow of participants through the trial is described (Fig. 2). Of note, we demonstrated success in all categories of feasibility where thresholds were pre-specified. We describe results for each feasibility question below, followed by results of emergent evidence of the potential effectiveness of TAPER.

**Table 3:** Participant characteristics

Variable	Intervention group (*n* = 18)	Control group (*n* = 19)
Age (in years); mean (SD)	79 (3.57)	80 (6.17)
Gender (female); *n* (%)	10 (56)	10 (53)
Income; *n* (%)		
≤ US $20,000	-	3 (16)
US $20,001–50,000	6 (33)	8 (42)
US $50,001–70,000	3 (17)	3 (16)
US $70,001–100,000	3 (17)	2 (11)
100,001–150,000	3 (17)	-
> US $150,000	-	1 (5)
Private insurance (yes); *n* (%)	1 (5.6)	1 (5)
BMQ general score; mean (SD)	20.17 (3.31)	21.37 (3.35)
*Range*	*15.00, 28.00*	*16.00, 27.00*
BMQ specific score; mean (SD)	30.35 (4.83)	31.47 (3.12)
*Range*	*17.00, 38.00*	*26.00, 38.00*
Charlson Comorbidity Index score; mean (SD)		
*Range*	3.61 (2.64) *1.00, 9.00*	2.95 (2.48) *0.00, 8.00*
Number of prescribed medications; mean (SD)		
*Range*	7.33 (1.78) *4.00, 10.00*	8.26 (3.63) *5.00, 18.00*

*M* mean, *SD* standard deviation, *BMQ* beliefs about medicines questionnaire, general score ranging from 8 to 40 with higher values indicating stronger beliefs that medicines are overused and may cause harm, and specific score ranging from 10 to 50 with higher values indicating stronger belief in the necessity of patient-specific medication use and more concerns regarding patient-specific medication use; Charlson Comorbidity Index score ranges 0–33 with higher values representing higher comorbidity

**Table 4:** Process feasibility results: enrollment and recruitment

Feasibility category	Outcome	Criteria for success	N (%)
Process	Number of participants invited, Number of participants enrolled	Not applicable 20% enrolled	85 39 (46% of invited)
	Number of participants lost to follow-up	Less than 20%	3 (8% of enrolled)
	Number of participant withdrawals	Less than 20%	2 (5% of enrolled)
	Pharmacist recruitment	50%	3 (100% of invited)
	Physician recruitment	50%	31 (100% of invited)
	Number of data collection visits	Not applicable	78

**Table 5:** Process feasibility results: eligibility, data collection, and unblinding

Feasibility category: process			
**Outcome**	**Criteria for success**	***N* (%)/description**	
Challenges with determining the extent to which participants meet eligibility criteria	Ability to identify and mitigate challenges that arise during recruitment and eligibility checking in larger trial	• The list of potential participants from the EMR was a snapshot in time; medication changes that occurred after this were not reflected, and some potential participants did not know if they were on > 5 log-term medications. *Mitigation*: the researcher confirmed participants were on 5 or more long-term medications during the initial recruitment phone call; this was then confirmed by the current medication list provided by their community pharmacy	
Nature of challenges in terms of participants’ understanding and ability to respond to the surveys	Ability to identify and mitigate challenges that arise during data collection appointments in larger trial	• One participant spoke English but had trouble with reading the answer options in English and concentrating throughout the length of the interview. *Mitigation*: a family member was engaged for help with translation for a participant of the answer options. Exclusion criteria will cover in main RCT • Hearing or vision impairments and fatigue in concentration made some interviews longer. *Mitigation*: questions and/or answer options were repeated carefully, and interviews could be split, breaks were taken during data collection appointments, or the appointment was broken into multiple, shorter sessions	
Number and nature of instances of unblinding	Less than 10 instances of unblinding; ability to identify and mitigate challenges that arise during instances of unblinding in larger trial	Occurrence (*n* = 6)	Follow-up action
		Study staff triggered unblinding: 1. Pharmacist had issue with TaperMD login and asked blinded research assistant assessing outcomes (BRAO) for assistance 2. Pharmacist showed message with patient’s name on it to the BRAO when they required assistance 3. Participant also called BRAO about problem with appointment scheduling after the BRAO had been unblinded	The operational manual was updated to note these examples of unblinding and amend processes to prevent including assigning roles not related to outcome assessment to other researchers to avoid contamination risk. These participants were included in analyses
		Participant triggered unblinding: 1. Participant told BRAO what occurred in terms of medication changes when called to set up 6-month data collection appointment 2. Question in health utilization questionnaire asks about visits to pharmacists, if participants tell BRAO about seeing a pharmacist and providing more details about seeing the pharmacist (versus just stating they saw a pharmacist) 3. Participant asks BRAO when they will be seeing the pharmacist and receiving the intervention when called to make 6-month data collection appointment	

*EMR* electronic medical record, *BRAO* blinded research assistant assessing outcomes

**Table 6:** Resource feasibility outcomes

Feasibility category	Outcome	Criteria for success	N/description
Resources	Length of time to complete the surveys	Less than 50% of participants require more than 1 visit to complete data collection; less than 2 h required for data collection visits	Baseline visit: 1 to 2.5 h 6-month visit: 1 to 1.5 h Two people required multiple visits due to fatigue with data collection
Resources	Travel time for the research team done to complete visits	Less than 50% of visits require ≥ 30 min of travel time (one way)	No travel time for office data collection Time to travel to participant’s house ranges from 10- to 30-min driving time one way Baseline: 27 visits at home, 13 office visits 6 months: 17 visits at home, 18 office visits

**Table 7:** Management feasibility outcomes

Feasibility category	Outcome	Criteria for success	Description
Management	Nature of data entry problems/changes	Ability to identify and mitigate common data entry errors (e.g., data missing at random, data syncing issues, problems with database) in larger trial	• Data was collected using a Microsoft Access database on the research computer. In 15 instances, the database did not launch property on the computer for the data collection visit. The researcher completed the surveys on paper with the participant and then entered the data once they returned to the office • The Manty questionnaire was not entered in its entirety in the Access database; only the 3 main questions were entered • During the final data synchronization from the Access database to Excel to complete the analysis, 16 entries were missing. The researcher used paper records to fill in the missing data fields • A measure of strength (hand grip) and 2 questionnaires (one assessing healthcare utilization, and a second assessing sleep derived from the 15 D quality-of-life questionnaire) were added midway through the study to pilot, so not all study participants had these outcomes measured at their baseline and 6-month data collection appointments • The Stanford self-efficacy questionnaire [61] was stopped after the first few baseline data collection appointment due to difficulties in administration and feedback from the participants and researcher. Results were not included in analysis or reported

### Process feasibility research questions

Responses generally came in within the first 2 weeks after mailing patients. We assessed 85 participants for eligibility, and 39 (46%) of these were enrolled into the study and subsequently randomized. Two participants (5%) withdrew from the trial (1 from the intervention group and 1 from the control group), and 3 (8%) were lost to follow-up (2 from the intervention group and 1 from the control group). Two participants were excluded from the intention-to-treat statistical analysis as they were under study inclusion criteria for age. All three pharmacists and 31 physicians who were invited to participate in this study agreed to take part. Overall, the structure of the intervention was considered efficient and fit with normal clinical workflow based on clinical user reports; the family physicians took no longer than the 30 min allotted and could sometimes be completed in less time. Furthermore, some participants did not actually start the deprescribing plan right after the physician appointment and did not implement recommendations as they believed there was a further step. In response to this, the process was adjusted to include a pharmacist check-in call with the patient a week after the physician appointment to reinforce the instructions of the plan and respond to any questions. Six instances of unblinding of the outcome assessor occurred. All the recruitment and randomization process outcomes met or exceeded the threshold for success stated a priori (Tables 4 and 5)*.*

We trialled two quality-of-life scores, and the general feedback was that the WHODAS was longer and more cumbersome to administer in this group [57]. Similarly, the Pittsburgh sleep scale [58] was lengthy considering it was not a primary outcome domain, and so we identified and tested a single item for sleep quality in the latter part of the trial, which was more practical to administer [62]. The Stanford Self-Efficacy Scale for Managing Chronic Disease and the Flinders Fatigue scale [50, 61] were abandoned early on as it was felt by both participants and study personnel to be too lengthy and difficult to administer.

### Resource feasibility questions

The processes for data collection fit with our pre-specified criteria for success. Fifty-nine percent of data collection visits were performed at the participant’s home, and none of these visits required a travel time of longer than 30 min one-way. Data collection appointments were able to be kept to less than 2 h long in most cases, with baseline visits taking 1.5–2 h and the 6-month visit 1–1.5 h. There were 2 instances where baseline data collection took up to 2.5 h. In response, we adjusted processes, allowing participants to break data collection appointments into multiple sessions if they became fatigued. Also pertinent to this, as outlined later in the results, section several questionnaires were felt by researchers and participants to be too taxing for the yield. All the data collection resource outcomes met or exceeded the threshold for success stated a priori (Table 6).

### Management feasibility questions

We identified several data management/entry errors that operational changes in data management would mitigate in a larger trial. Data management and entry errors included accidental exclusion of survey questions from the database and problems with launching and syncing of the database. We were unable to calculate scores for the role-emotional domain as there was misalignment between our database and SF36-V1 response options for these questions when the database was programed, and bodily pain domain as these questions were omitted from the database, and thus, this data was not collected.

Database launching and syncing errors were mitigated by utilizing paper surveys as a backup, and exclusion of questions will be corrected by ensuring the database is correctly set up prior to implementation of a larger trial. Three additional outcome measures were also added into the study protocol for testing midway through the trial: hand grip strength, the healthcare utilization questionnaire, and the 15D quality-of-life sleep question, so these outcomes were not measured for most participants at baseline, and also not measured for several participants at 6 months. All the data management outcomes met or exceeded the threshold for success stated a priori (Table 7).

### Scientific feasibility questions

Collecting data on potential outcome measures allowed us to see the range and variance in the target group and to assess and compare tools both in their variance and in the practicality of their administration in this group. The detailed results of these outcomes can be found in Tables 8 and 9, Tables 10–14 in Additional file 1, and results are presented visually in Figs. 3–7 in Additional file 4. We found no substantive floor or ceiling effects. With such a small sample size, higher standard deviations are to be expected and should be reduced in a large-scale trial. We found nothing to preclude use of these instruments in a larger trial, except for a few based strictly on feasibility in terms of process (Tables 8, 9, 10).

**Table 8:** Number of prescribed, prescribable, and non-prescribable medication at 6 months by group

6-month number of medications	Intervention	Control
Prescribed medications^a^; mean (SD)	7.00 (1.75)	7.65 (2.83)
Prescribable medications^a^; mean (SD)	2.00 (1.41)	1.88 (1.45)
Non-prescribable medications; mean (SD)	1.13 (1.26)	1.24 (1.98)

*SD* standard deviation

^a^Proposed primary outcomes for larger trial

**Table 9:** Number of medication changes in intervention arm patients (*n* = 18) during the 6-month study period

Medication change	Prescribed *n* (%)	Prescribable *n* (%)	Non-prescribable *n* (%)
Medications stopped^a^	11 (61)	6 (33)	4 (22)
Medications started^b^	7 (39)	1 (6)	1 (6)
Medications switched^c^	5 (28)	0 (0)	0 (0)
Medications with dose reduction^d^	5 (28)	1 (6)	0 (0)
Medications with dose increase^e^	9 (50)	0 (0)	0 (0)
Medications failed taper^f^	6 (33)	0 (0)	1 (6)

^a^A medication was discontinued

^b^A new medication was started

^c^A medication was switched to a different medication, considered to be safer, as a direct result of TAPER

^d^A medication was reduced in dose or frequency of administration

^e^A medication was increased in dose or frequency of administration

^f^A medication was “paused and monitored” but restarted within the 6-month study period due to reoccurrence of symptoms or otherwise clinically indicated

**Table 10:** Feasibility questions and the classification of the feasibility sub-questions

**1. To what extent is implementation of TAPER feasible in a primary care setting?**	**Categories of outcomes**
What is the recruitment, refusal, and drop-out rate of participants, pharmacists, and family physicians?	Process
What are the challenges with determining to extent to which participants meet eligibility criteria?	Process
What is the nature of challenges in terms of participants’ understanding of, and ability to respond to, the surveys?	Process
What is the nature of any instances of unblinding?	Process
What is the length of time to complete the surveys?	Resources
How much travel time does the research team do to complete visits?	Resources
What is the nature of data entry/database problems?	Management
What is the nature of any serious adverse events associated with the intervention or study process?	Scientific
What is the variance, potential floor, and ceiling effects for research outcomes?	Scientific
**2. Is there any emergent evidence of direction of effect of TAPER compared to usual care on a range of potential outcomes?**	
Outcomes include number of medications (potential primary outcome for larger randomized controlled trial), medication dose, quality of life, psychological distress, cognition, mobility-related fatigue, nutritional status, level of mobility functioning, sleep quality, patient enablement, grip strength, falls, healthcare utilization, and adverse events	

### Emergent evidence of potential effectiveness

While we were not assessing for significant differences between groups at 6 months as this study was not designed with adequate power to do this, and a number of adjustments to process were made, we examined the data for signals around the direction of effect of outcome measures (Figs. 3–7, Additional file 4). Our results do show that most of the outcome measures signal a direction towards the effectiveness of TAPER compared to usual care, with the exception of the mental health domain of the SF-36, patient enablement, MMSE, and grip strength. There were no meaningful differences between groups for serious adverse events, and the intervention itself was not associated with any serious adverse events (Tables 12 and 13, Additional file 1).

### Sensitivity analysis

The results of the sensitivity analyses using per-protocol approach are provided in the additional information file. The overall conclusion for all the outcomes was similar to the primary analyses.

## Discussion

We examined the extent to which TAPER is feasible to implement in a primary care setting and if there is any emerging evidence of the direction of effect of TAPER compared to usual care on a range of outcomes. We found support that the trial met all pre-specified thresholds for success across all feasibility indicators. Notably, 100% of invited pharmacists and family physicians participated, 46% of patients assessed for eligibility were enrolled, and the number of participants who withdrew was low, with reasons unrelated to the study (e.g., surgery). These results exceeded our thresholds for success and give us confidence for a larger trial where uptake and participation are essential.

Operationally, research procedures were relatively efficient. The number of participants lost to follow-up was low, and the primary outcome could still be ascertained in those who were unavailable for the full outcome assessment. Furthermore, instances of unblinding, which were balanced between staff versus participant, triggered unblinding, and challenges identified are those easily remediated in a larger trial with more staff and with clear role assignment among the research team. Travel time (10–30 min) and time to complete data collection were reasonable (1–2.5 h); generally, only one visit was required to collect data at baseline or follow-up. However, in few instances, more time or splitting collection up over two visits was required. These strategies were particularly relevant for those participants whose primary language was not English or had hearing or vision problems. Data management issues relating to using Microsoft Access software to enter some of the scales and entering in multiple and remote locations were significant enough to search for alternate software to use for a future larger randomized controlled trial. The intervention itself was not associated with any serious adverse events, confirming findings from a previous study [59].

With a few exceptions, we also found evidence to support our hypothesized group differences. Even in this small sample size, the groups were reasonably similar in demographic makeup, with a slightly higher Charlson comorbidity burden and a slightly lower number of prescribed medications in the intervention versus control group at baseline. Data were inadequate to make meaningful conclusions about any emergent evidence of potential effectiveness on healthcare utilization, with emergency department visits or hospitalizations occurring too infrequently in such a small sample. Furthermore, meaningful conclusions about cognition are also challenging given that most people in the study had MMSE scores between 26 and 30. This is not surprising given that we excluded people who did not have adequate cognitive skills to understand and respond to the surveys. We did however find that the outcome measures we included in this trial showed no evidence of floor or ceiling effects and generally performed such that with a larger sample and adequate power; we expect to be able to make conclusions about the effectiveness of TAPER on these outcomes. Only 5 participants, out of 37, had missing data in this pilot trial. We implemented multiple imputation approach to impute these missing data based on this small sample size. Thus, this multiple imputation had some impact on the precision of the estimated treatment effect.

Together, our results provide the support needed to proceed with a full randomized controlled trial, with some modifications, so on the basis of these data, we made the decision to proceed with a larger RCT. These findings allowed us to shorten the study data collection by providing a sound basis for refining the number and choice of outcome measures, reduce the risks of unblinding of outcome assessors, and seek new data management software. The signals of effect also support proceeding with formal testing of the hypothesis that TAPER will reduce medications, and that the negative associations of polypharmacy with health outcomes may be at least partly reversible if this is achieved.

## Supplementary Information

**Additional file 1:** Detailed description of outcomes. **Table 10.** Primary and secondary outcome measures using intention-to-treat approach. **Table 11.** Number (%) of participants experiencing falls and healthcare utilization. **Table 12.** Number (%) of participants experiencing a serious adverse event. **Table 13.** Serious adverse events description. **Table 14.** Number (%) of participants experiencing changes in side effects.

**Additional file 2.** Consort extension for pilot and feasibility trials checklist.

**Additional file 3.** TIDier Checklist.

**Additional file 4:** Results of patient outcome measures. **Fig. 3.** EQD5 and SF36-V1 quality of life scales. **Fig. 4.** WHODAS, psychological distress, mobility fatigue, sleep quality. **Fig. 5.** Patient enablement, cognition, and nutrition. **Fig. 6.** Number of medications. **Fig. 7.** Side effects at 6-months.

**Additional file 5.** List of machine screen flags within TaperMD.

## Data Availability

The study team will have full access to the dataset. All data analyzed during the current study will be available from the corresponding author on reasonable request once all planned analyses and publications by the study team are complete. Anonymized patient level data will be made available for meta-analyses, and participant consent forms are available upon reasonable request. Access to the statistical code will not be granted.

## Abbreviations

BRAO: Blinded research-assistant assessing outcomes
EMR: Electronic medical record
Health TAPESTRY: Health Teams Advancing Patient Experience: Strengthening Quality
MMSE: Mini-mental status examination
RCT: Randomized controlled trial
SF-36-V1: Short-Form Health Survey, 36-item
TAPER: Team approach to polypharmacy evaluation and reduction
WHODAS: World Health Organization Disability Assessment Schedule
