# Supplementary material for: Team approach to polypharmacy evaluation and reduction: feasibility randomized trial of a structured clinical pathway to reduce polypharmacy
Source: Pilot Feasibility Stud. 2023 May 18;9:84. doi: 10.1186/s40814-023-01315-0 (PMC10193598; doi:10.1186/s40814-023-01315-0)
Supplement: Supplementary file 1 — Additional file 1: Detailed description of outcomes. Table 10. Primary and secondary outcome measures using intention-to-treat approach. Table 11. Number (%) of participants experiencing falls and healthcare utilization. Table 12. Number (%) of participants experiencing a serious adverse event. Table 13. Serious adverse events description. Table 14. Number (%) of participants experiencing changes in side effects. [file 40814_2023_1315_MOESM1_ESM.pdf]

**Additional File 1: Detailed description of outcomes**

**Table 10: Primary and secondary outcome measures using intention-to-treat approach (Imputing Missing Data)**

| **Outcome** | **Intervention (n=18)** | | **Control (n=19)** | | **Mean difference or Odds ratio (95% CI)** | **Mean difference or Odds ratio (95% CI)** | **Hypothesis support** |
|---|---|---|---|---|---|---|---|
| | **Baseline** | **6-Month** | **Baseline** | **6-Month** | **ITT (n=37)** | **Per-protocol (n=32)** | |
| Number of medications (prescribed and prescribable); Mean (SD) *Range* | 7.39 (1.75) *(4, 10)* | 9.00 (2.28) *(6, 14)* | 8.26 (3.63) *(5, 18)* | 9.53 (3.28) *(5, 19)* | RR=0.97 (0.76, 1.23) p=0.781 | RR=0.97 (0.77, 1.21) p=0.761 | ✓ |
| Number of medications (prescribed); Mean (SD) *Range* | 7.33 (1.78) *(4.00, 10.00)* | 7.00 (1.75) *(4.00, 10.00)* | 8.26 (3.63) *(5.00, 18.00)* | 7.65 (2.83) *(5.00, 16.00)* | RR=0.90 (0.69, 1.16) p=0.415 | RR=0.94 (0.72, 1.21) p=0.644 | ✓ |
| Quality of life (EQ5D-5L; M (SD)) *Range* | 0.82 (0.08) *(0.63, 0.95)* | 0.83 (0.08) *(0.69, 0.95)* | 0.75 (0.16) *(0.32 0.95)* | 0.75 (0.17) *(0.33, 0.95)* | 0.06 (-0.05, 0.17) p=0.245 | 0.09 (-0.02, 0.19) p=0.098 | ✓ |
| Quality of life (SF36; M (SD)) | | | | | | | |
| Physical functioning *Range* | 62.13 (18.32) *(33.30, 95.00)* | 56.67 (23.35) *(20.00, 100.00)* | 57.11 (26.99) *(15.00, 95.00)* | 49.33 (31.56) *(5.00, 100.00)* | 2.39 (-22.25, 27.03) p=0.840 | 7.81 (-13.72, 29.34) p=0.463 | ✓ |
| Role-Physical *Range* | 4.21 (0.66) *(3.00, 5.00)* | 3.84 (0.99) *(1.00, 5.00)* | 3.35 (1.11) *(1.80, 5.00)* | 3.49 (1.17) *(1.50, 5.00)* | 0.30 (-0.54, 1.14) p=0.471 | 0.32 (-0.52, 1.16) p=0.439 | ✓ |
| Vitality *Range* | 59.44 (23.19) *(15.00, 90.00)* | 57.33 (16.68) *(20.00, 85.00)* | 48.95 (19.76) *(15.00, 85.00)* | 51.88 (24.62) *(0.00, 90.00)* | 4.36 (-20.15, 28.87) p=0.695 | 4.91 (-11.19, 21.02) p=0.537 | ✓ |
| Mental health *Range* | 84.67 (12.89) *(56.00, 96.00)* | 83.20 (14.45) *(48.00, 100.00)* | 82.74 (11.55) *(60.00, 96.00)* | 85.50 (10.32) *(56.00, 100.00)* | -3.20 (-18.43, 12.03) p=0.639 | -1.21 (-10.47, 8.04) p=0.790 | ✗ |
| Social functioning *Range* | 92.36 (12.96) *(62.50, 100.00)* | 94.17 (14.07) *(50.00, 100.00)* | 78.95 (26.70) *(12.50, 100.00)* | 85.16 (20.52) *(25.00, 100.00)* | 8.20 (-7.63, 24.03) P=0.294 | 8.59 (-4.89, 22.07) p=0.202 | ✓ |
| General health *Range* | 68.61 (15.70) *(35.00, 90.00)* | | 64.21 (20.16) | | 5.20 (-11.86, 22.26) p=0.514 | 7.84 (-4.77, 20.44) p=0.214 | ✓ |

| Outcome | Intervention (n=18) | | Control (n=19) | | Mean difference or Odds ratio (95% CI) | Mean difference or Odds ratio (95% CI) | Hypothesis support |
|---|---|---|---|---|---|---|---|
| | **Baseline** | **6-Month** | **Baseline** | **6-Month** | **ITT (n=37)** | **Per-protocol (n=32)** | |
| | | 71.67 (13.18) *(55.00, 95.00)* | *(15.00, 90.00)* | 63.59 (19.15) *(30.00, 100.00)* | | | |
| Quality of life (WHODAS; M (SD)) *Range* | 29.03 (7.77) *(18.00, 45.56)* | 27.86 (7.09) *(18.33, 41.67)* | 33.16 (9.48) *(20.00, 56.67)* | 29.97 (12.44) *(13.89, 60.00)* | 0.01 (-8.71, 8.72) p=0.993 | -2.36 (-10.39, 5.67) p=0.552 | No difference |
| Psychological distress; M (SD) *Range* | 12.56 (2.96) *(9.00, 20.00)* | 12.20 (2.83) *(9.00, 19.00)* | 13.42 (2.73) *(9.00, 19.00)* | 14.75 (3.64) *(9.00, 21.00)* | -2.05 (-4.86, 0.77) p=0.146 | -2.46 (-4.96, 0.03) p=0.053 | ✓ |
| Cognition; M (SD) *Range* | 28.17 (1.89) *(24.00, 30.00)* | 28.73 (1.67) *(25.00, 30.00)* | 27.63 (2.83) *(18.00, 30.00)* | 28.69 (2.47) *(21.00, 30.00)* | -0.24 (-1.89, 1.42) p=0.774 | 0.10 (-1.52, 1.71) p=0.902 | ✗ |
| Severe impairment (0-9) | - | - | - | - | | | |
| Moderate impairment (10-19) | - | - | 1 (5.3) | - | | | |
| Mild impairment (20-25) | 2 (11.1) | 1 (5.6) | 1 (5.3) | 2 (10.5) | | | |
| Potentially normal (26-30) | 16 (88.9) | 14 (77.8) | 17 (89.5) | 14 (73.7) | | | |
| Mobility-related fatigue; M (SD) *Range* | 1.50 (1.15) *(0.00, 4.00)* | 1.40 (1.24) *(0.00, 4.00)* | 1.74 (1.66) *(0.00, 6.00)* | 1.75 (1.73) *(0.00, 5.00)* | -0.36 (-1.62, 0.95) p=0.589 | -0.46 (-1.60, 0.67) p=0.408 | ✓ |
| Nutritional status; M (SD) *Range* | 10.67 (1.28) *(7.00, 13.00)* | 10.13 (2.31) *(5.00, 13.00)* | 9.89 (1.94) *(6.00, 13.00)* | 10.00 (1.21) *(7.00, 11.00)* | 0.34 (-1.17, 1.85) p=0.641 | -0.07 (-1.36, 1.22) p=0.911 | ✓ |
| Mobility functioning; n (%) Walking 2.0km | | | | | N/A | N/A | N/A |
| Able to manage without difficulty | 4 (22.2) | 5 (27.8) | 7 (36.8) | 3 (15.8) | | | |
| Able to manage with some difficulty | 8 (44.4) | 6 (33.3) | 3 (15.8) | 5 (26.3) | | | |
| Able to manage with a great deal of difficulty | 2 (11.1) | 1 (5.6) | 2 (10.5) | 2 (10.5) | | | |
| Able to manage with the help of another person | 1 (5.6) | - | 1 (5.3) | 2 (10.5) | | | |

| Outcome | Intervention (n=18) | | Control (n=19) | | Mean difference or Odds ratio (95% CI) | Mean difference or Odds ratio (95% CI) | Hypothesis support |
|---|---|---|---|---|---|---|---|
| | **Baseline** | **6-Month** | **Baseline** | **6-Month** | **ITT (n=37)** | **Per-protocol (n=32)** | |
| Unable to manage | 3 (16.7) | 3 (16.7) | 5 (26.3) | 5 (26.3) | | | |
| Walking 0.5km | | | | | | | |
| Able to manage without difficulty | 12 (66.7) | 7 (38.9) | 10(52.6) | 10(52.6) | | | |
| Able to manage with some difficulty | 2 (11.1) | 6 (33.3) | 2 (10.5) | 2 (10.5) | | | |
| Able to manage with a great deal of difficulty | 1 (5.6) | - | 1 (5.3) | 2 (10.5) | | | |
| Able to manage with the help of another person | - | - | 2 (10.5) | - | | | |
| Unable to manage | 3 (16.7) | 2 (11.1) | 3 (15.8) | 3 (15.8) | | | |
| Climbing one flight of stairs | | | | | | | |
| Able to manage without difficulty | 11(61.1) | 9 (50.0) | 9 (47.4) | 8 (42.1) | | | |
| Able to manage with some difficulty | 6 (33.3) | 5 (27.8) | 8 (42.1) | 3 (15.8) | | | |
| Able to manage with a great deal of difficulty | - | - | - | 5(26.3) | | | |
| Able to manage with the help of another person | - | - | - | - | | | |
| Unable to manage | 1 (5.6) | 1 (5.6) | 1 (5.3) | 1(5.3) | | | |
| Sleep quality (15D item); n (%) | No baseline data | | No baseline data | | | | N/A |
| I am able to sleep normally | | 2 (11.1) | | 2 (10.5) | | | |
| I have slight problems with sleeping | | 3 (16.7) | | 5 (26.3) | | | |
| I have moderate problems with sleeping | | - | | 1 (5.3) | | | |
| | | 2 (11.1) | | - | | | |

| Outcome | Intervention (n=18) | | Control (n=19) | | Mean difference or Odds ratio (95% CI) | Mean difference or Odds ratio (95% CI) | Hypothesis support |
|---|---|---|---|---|---|---|---|
| | **Baseline** | **6-Month** | **Baseline** | **6-Month** | **ITT (n=37)** | **Per-protocol (n=32)** | |
| I have great problems with sleeping<br>I suffer severe sleeplessness | | - | | - | | | |
| Sleep (Pittsburgh sleep quality)<br>*Range* | 6.72 (3.44)<br>*(2.00, 14.00)* | 7.17 (3.55)<br>*(2.00, 12.00)* | 6.16 (3.82)<br>*(2.00, 15.00)* | 8.37 (3.67)<br>*(1.00, 15.00)* | -1.20 (-3.62, 1.22) p=0.319 | -1.14 (-3.65, 1.37) p=0.361 | ✓ |
| Patient enablement; M (SD)<br>*Range* | 5.53 (3.37)<br>*(0.00, 12.00)* | 4.21 (3.68)<br>*(0.00, 12.00)* | 5.33 (4.27)<br>*(0.00, 12.00)* | 4.75 (3.30)<br>*(0.00, 12.00)* | -1.02 (-3.91, 1.86) p=0.472 | -0.83 (-3.48, 1.83) p=0.528 | ✗ |
| Grip strength average† (in kg); M (SD) | No baseline data | | No baseline data | | | | N/A |
| Right hand<br>*Range* | | 22.78 (7.11)<br>*(14.00, 33.67)* | | 24.50 (8.45)<br>*(14.00, 38.67)* | -1.42 (-10.17,7.32) p=0.714 | -4.27 (-15.36, 6.83) p=0.401 | |
| Left hand<br>*Range* | | 21.33 (8.98)<br>*(10.67, 34.67)* | | 24.17 (9.17)<br>*(12.67, 38.67)* | -0.79 (-15.68, 14.10) p=0.891 | -5.80 (-18.51, 6.91) p=0.323 | |

M=mean, SD=standard deviation, RR=relative risk; SF36=The Short Form-36 Health Survey, WHODAS=World Health Organization Disability Assessment Schedule; Range included as scientific feasibility outcome to assess variance; EQ5D-5L range -0.148 to 0.949, higher scores represent higher quality of life; SF36 scores range 0-100 (except Role-Physical, range 1-5), higher scores represent higher quality of life for the domain; WHODAS range 0-144, higher scores represent higher disability; psychological distress range 9-45, higher scores represent higher psychological distress; cognition range 0-30, with lower scores representing higher impairment; mobility-relate fatigue scores range 0-6, with higher scores representing higher fatigue; nutrition range 0-14, higher scores represent better nutritional status; sleep quality range 0-21, higher scores represent poorer sleep quality; patient enablement range 0-12, higher scores represent higher patient enablement; grip strength higher scores represent higher strength. Mobility functioning and sleep (15D) had sufficient missing data to preclude calculation of effect.

† An outlier was excluded from analysis of grip strength.

✓ Supports hypothesized difference

✗ Does not support hypothesized difference

N/A Where new scales of items were introduced part way through the trial for testing (for example where it was apparent other measures were unwieldy) they are not recorded in the hypothesis testing assessment

**Table 11: Number (%) of participants experiencing falls and healthcare utilization**

| Outcome | Intervention (n=18) | | Control (n=19) | |
|---|---|---|---|---|
| | Baseline | 6-Month | Baseline | 6-Month |
| Falls, n (%) | | | | |
| No fall | 13 (72.2) | 14 (77.8) | 14 (73.7) | 16 (84.2) |
| 1 fall | 3 (16.7) | 3 (16.7) | 4 (21.1) | 2 (10.5) |
| Multiple falls (2+) | 2 (11.1) | 1 (5.6) | 1 (5.3) | 1 (5.3) |
| Falls requiring healthcare, n (%) | (n=5) | (n=4) | (n=5) | (n=3) |
| None required | 2 (40.0) | 3 (75.0) | 1 (20.0) | 1 (33.3) |
| Some required | 3 (60.0) | 1 (25.0) | 4 (80.0) | 2 (66.7) |
| Healthcare utilization, n (%) | | | | |
| ED visits | 1 (5.6) | 6 (33.3) | 2 (10.5) | 2 (10.5) |
| Urgent care visits | 1 (5.6) | 2 (11.1) | - | 2 (10.5) |
| Total ED/Urgent care visits | 2 (11.1) | 8 (44.4) | 2 (10.5) | 4 (21.1) |
| Hospital admissions | - | 2 (11.1) | 1 (5.3) | 2 (10.5) |
| Primary care visits | 11 (61.1) | 17 (94.4) | 13 (68.4) | 16 (84.2) |
| Long-term care admissions | - | - | - | - |
| Specialist visits | 8 (44.4) | 14 (77.8) | 9 (47.4) | 9 (47.4) |
| Homecare services | - | - | 1 (5.3) | 1 (5.3) |
| Homecare visits | - | - | - | - |
| Professional care services | 6 (33.3) | 10 (55.6) | 5 (26.3) | 9 (47.4) |
| Supporting equipment use | 1 (5.6) | 1 (5.6) | 1 (5.3) | 1 (5.3) |
| Caregiver support | 3 (16.7) | 3 (16.7) | 2 (10.5) | 1 (5.3) |
| ED visits; Mean (SD) *Range* | - | 1.00 (0.00) *(1.00, 1.00)* | 1.00 (0.00) *(1.00, 1.00)* | 1.00 (0.00) *(1.00, 1.00)* |
| Urgent care visit; Mean (SD) *Range* | 1 (0) *(1,1)* | 1 (0) *(1,1)* | - | 1 (0) *(1,1)* |
| Total ED/Urgent care visits *Range* | 1 (0) *(1,1)* | 1 (0.41) *(1,2)* | 1 (0) *(1,1)* | 1 (0.58) *(1,2)* |
| Hospital admissions *Range* | - | 1 (0.71) *(1, 2)* | 1 (0) *(1,1)* | 1 (0) *(1,1)* |
| Primary care visits *Range* | 1.46 (2.16) *(1.00, 2.00)* | 3.18 (3.15) *(1.00,10.00)* | 2.77 (2.64) *(1.00, 8.00)* | 3.13 (2.22) *(1.00, 8.00)* |

| | | | | |
|---|---|---|---|---|
| Specialist visits<br>*Range* | 1.25 (0.46) *(1.00, 2.00)* | 2.21 (0.97) *(1.00, 4.00)* | 1.67 (0.71) *(1.00, 3.00)* | 1.67 (0.50) *(1.00, 2.00)* |
| Professional care services<br>*Range* | 2.00 (1.27) *(1.00, 4.00)* | 1.80 (1.40) *(1.00, 5.00)* | 2.2 (1.79) *(1.00, 5.00)* | 1.22 (0.44) *(1.00, 2.00)* |

Baseline healthcare utilization items are within past 3 months, 6-month healthcare utilization is 'since baseline data collection'. ED = emergency department. SD = standard deviation.

**Table 12: Number (%) of participants experiencing a serious adverse event**

| **Event** | **Time point** | **Intervention (n=18)** | **Control (n=19)** |
|---|---|---|---|
| Serious adverse event, n (%) | Week 1 | 0 (0) | 0 (0) |
| | Month 3 | 1 (5.6) | 0 (0) |
| | Month 6 | 0 (0) | 2 (10.5) |
| Serious adverse event determined to be related to TAPER[1], n (%) | Week 1 | 0 (0) | 0 (0) |
| | Month 3 | 0 (0) | 0 (0) |
| | Month 6 | 0 (0) | N/A |

[1]For each serious adverse event reported, the Principal Investigator (a physician) determined whether the incident was related to the TAPER intervention. N/A=not applicable as the control group did not receive TAPER

**Table 13: Serious adverse events description**

| **Group** | **Week 1** | **Month 3** | **Month 6** |
|---|---|---|---|
| TAPER | None | 1. Painful calf and foot, operated on two blood clots; pain in foot gone now [ED visit for right leg ischemia resulting in hospitalization, operation to remove two blood clots] | None |
| Control | None | None | 1. Thought I had a kidney stone, turned out to be an aneurism [ED visit for flank pain, diagnosed with renal artery aneurism]<br>2. Stroke in April [ED visit and subsequent hospitalization for ischemic stroke] |

ED=emergency department

**Table 14: Number (%) of participants experiencing changes in side-effects**

| Variable | Intervention (n=18) | Control (n=19) |
|---|---|---|
| Any symptoms or side effects stopped; n (%) | 4 (22.2) | 2 (10.5) |
| Any symptoms or side effects improved; n (%) | 7 (38.9) | 10 (52.6) |
| Any symptoms or side effects started; n (%) | 5 (27.8) | 4 (21.1) |
| Any symptoms or side effects increased; n (%) | 4 (22.2) | 7 (36.8) |
| Experienced any other health improvements from changing your medications; n (%) | 2 (11.1) | 8 (42.1) |
| Experienced any other problems since changing your medications; n (%) | 5 (27.8) | 9 (47.4) |
