# Supplementary material for: Team approach to polypharmacy evaluation and reduction: feasibility randomized trial of a structured clinical pathway to reduce polypharmacy
Source: Pilot Feasibility Stud. 2023 May 18;9:84. doi: 10.1186/s40814-023-01315-0 (PMC10193598; doi:10.1186/s40814-023-01315-0)

Additional File 4: Visual representations of the patient outcome measures

Figure 3. EQD5 and SF36-V1 quality of life scales

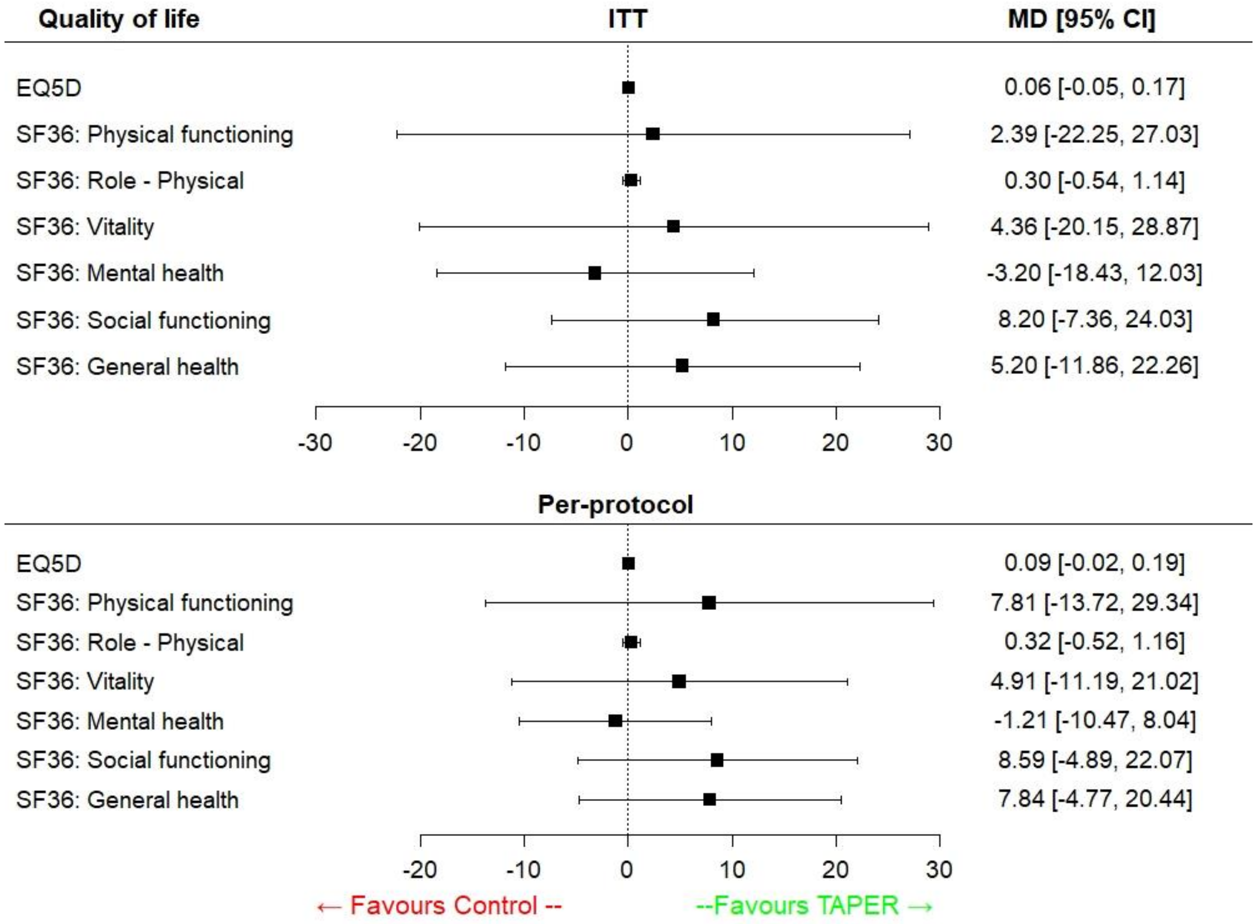

Figure 4. WHODAS, psychological distress, mobility fatigue, sleep quality

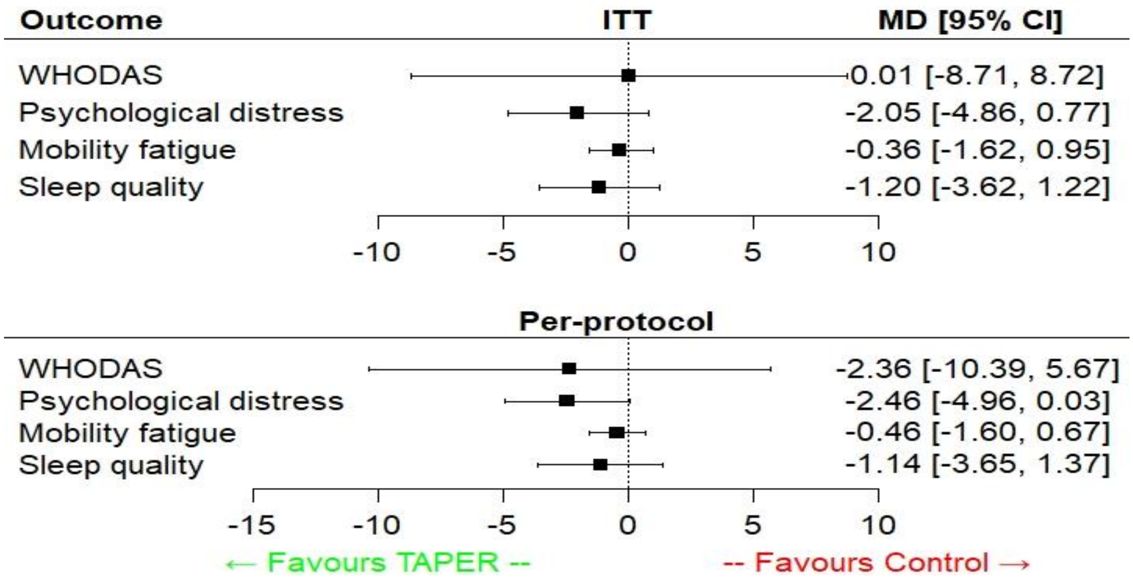

Figure 5. Patient enablement, cognition, and nutrition

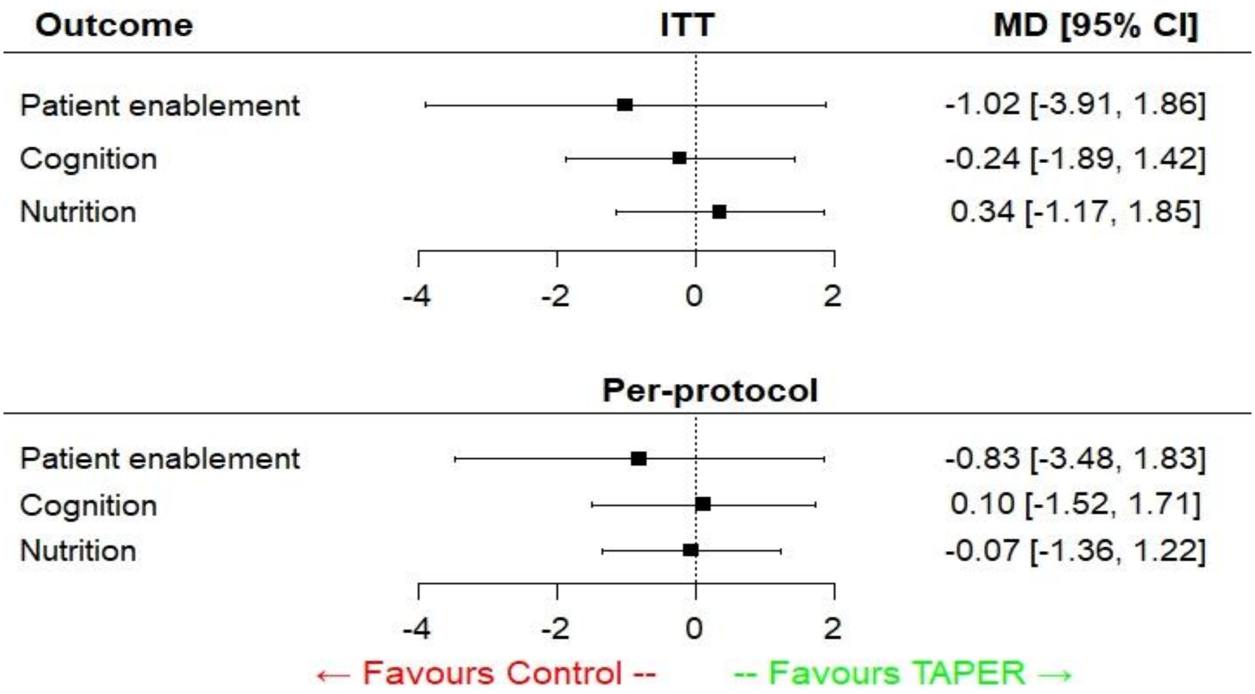

Figure 6. Number of medications

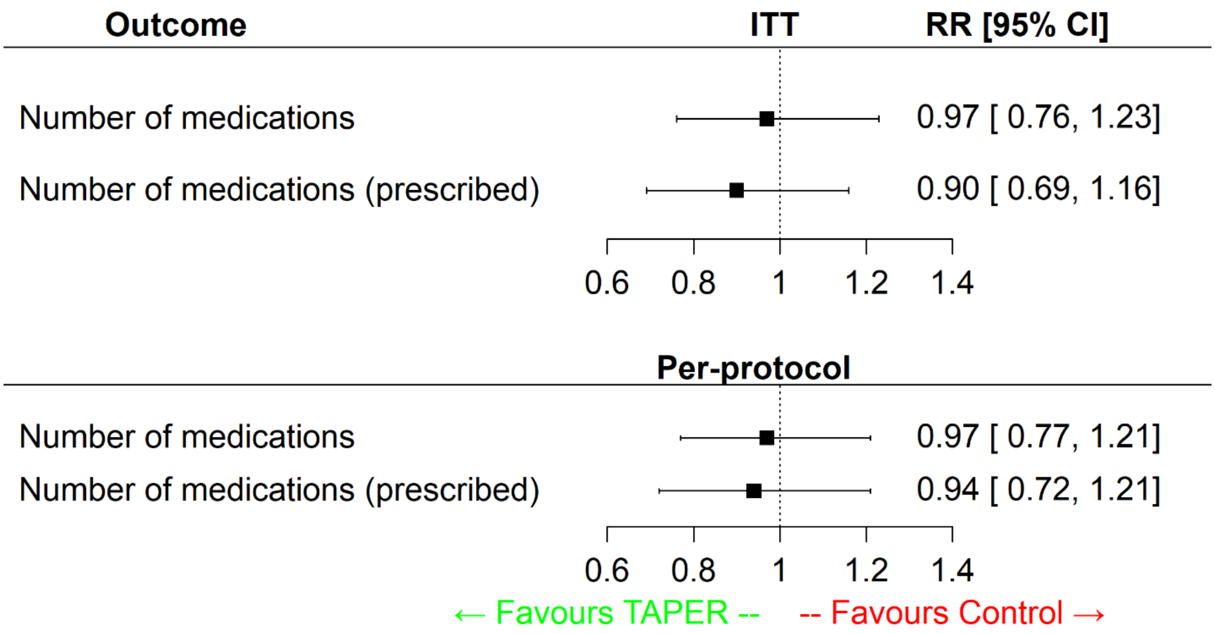

Figure 7. Side effects at 6-months

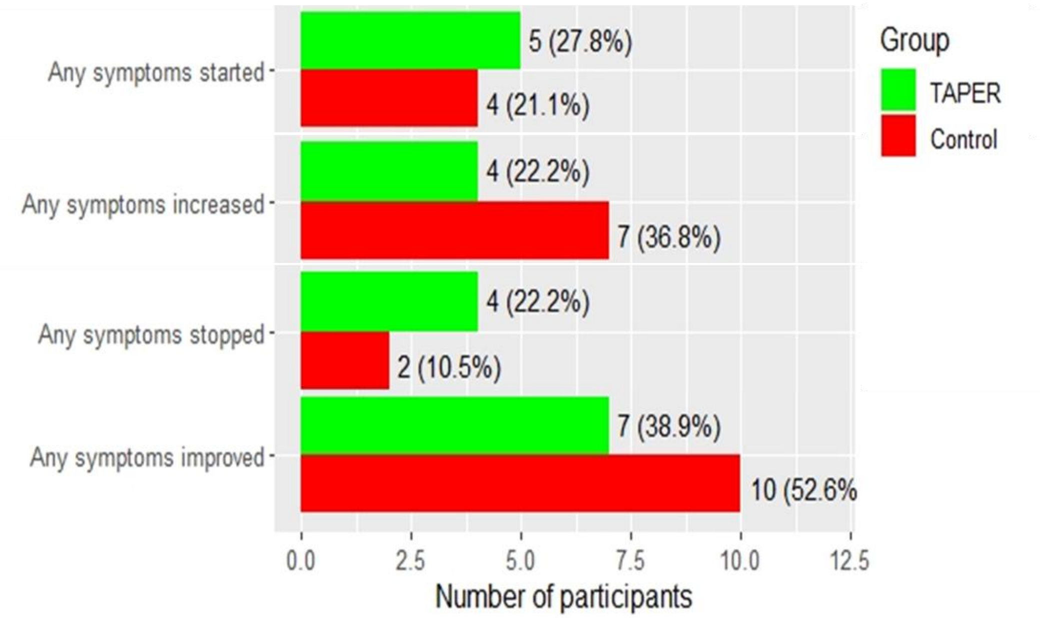

Supplement: Supplementary file 4 — Additional file 4: Results of patient outcome measures. Fig. 3. EQD5 and SF36-V1 quality of life scales. Fig. 4. WHODAS, psychological distress, mobility fatigue, sleep quality. Fig. 5. Patient enablement, cognition, and nutrition. Fig. 6. Number of medications. Fig. 7. Side effects at 6-months. [file 40814_2023_1315_MOESM4_ESM.pdf]
