# Supplementary material for: Team approach to polypharmacy evaluation and reduction: feasibility randomized trial of a structured clinical pathway to reduce polypharmacy
Source: Pilot Feasibility Stud. 2023 May 18;9:84. doi: 10.1186/s40814-023-01315-0 (PMC10193598; doi:10.1186/s40814-023-01315-0)
Supplement: Supplementary file 5 — Additional file 5. List of machine screen flags within TaperMD. [file 40814_2023_1315_MOESM5_ESM.pdf]

Additional File 5: List of machine screen flags within TaperMD

A machine screen flags potentially inappropriate medicines in older adults, or which carry a higher risk of medication adverse events, interactions and black box warnings is provided for those medications entered. This machine screen is supported by evidence including:

1. Potentially Inappropriate Medicines, from an environmental scan of jurisdictional lists (1-3), with checking against evidence sources, emerging evidence and newly registered drugs every 6 months. This list has been made open access as part of a collaboration with the American Society of Consultant Pharmacists at PIMsplus.org.
2. Drug-drug interaction checker (Wolters Kluwer existing product)
3. Anticholinergic burden: Anticholinergic medications may contribute to events such as fall, delirium, and cognitive impairment in older patients. There are a number of anticholinergic burden scoring systems. A systematic review was published in 2015 (4). After personal communication with the lead author of the systematic review we have chosen to include a table of scores from the article by Boustani M, et al. which has been the most extensively validated as a predictor of clinically important (5).
4. Warnings of potential 'drugs to avoid' or Black Box warnings (FDA, Prescrire International). FDA Black Box Warning used as Health Canada do not provide a database of structured monographs that allow electronic access to warnings. This will be flagged to clinicians using the tool. At present only PDFs of individual product monographs are accessible on a drug-by-drug basis, and in a format that is not searchable. We approached Health Canada repeatedly about accessing a searchable form of boxed warnings to link and have received no positive response. When this data is made available in a usable format it will be added to the tool.
5. Serotonergic drug burden (TaperMD proprietary product developed for study): Drugs that increase serotonin levels have an additive effect and serotonin syndrome is a predictable consequence of excess serotonin on the nervous system. Symptoms include cognitive effects, autonomic, and somatic effects and may range from barely perceptible to fatal. The syndrome is produced most often by the concurrent use of two or more drugs that enhance central nervous system serotonin activity. It often goes unrecognized because of the varied and nonspecific nature of its clinical symptoms, and is increasingly important as prescription of drugs such as selective serotonin reuptake inhibitor (SSRI) antidepressants for depression and tramadol for pain are more common: the proportion of the population on SSRI antidepressants is now around 10%, and the rise in prescribing is largely due to increasing long-term maintenance prescription. Serotonin syndrome manifests in alterations in cognition (disorientation, confusion), behaviour (agitation, restlessness), autonomic nervous system function (fever, shivering, sweating, diarrhea), and neuromuscular (ataxia, hyperreflexia, myoclonus) activity. These kinds of subtle effects may be easily missed or misattributed in an older adult (6).
    a. Serotonin syndrome: We have developed a scale for the study, licensed under creative commons, that flags those drugs that either alone in high dose or in combination can produce serotonin syndrome.
6. QT interval prolonging drug burden: Some drugs prolong the QT interval on a patient's electrocardiogram and thereby increase the risk of torsades de pointes (TdP), a heart

arrhythmia that can cause sudden cardiac death. We license and use the list provided and maintained at CredibleMeds® by the Arizona Center for Education and Research on Therapeutics (AZCERT), an independent non-profit organization. TaperMD uses this list to flag drugs with potential QT interval problems.

7. Hypotensive drug burden (TaperMD proprietary product developed for study). Rationale: Medications that cause hypotension may increase fall risk, and cause symptoms such as fatigue, dizziness. Drugs causing hypotension are one of the main risk groups for admission to hospital for adverse drug reactions in older adults. The aims of treatment are higher in older age and doses may need reducing. It is always useful to trial pause and monitor in patients who have been normotensive with long term treatment, to test the presence of hypertension, as studies show many can be successfully discontinued with no return of the original indication. We developed a scale for the study that flags drugs that potentially cause hypotension. This scale is licensed under creative commons.

References

1. Gallagher P, Ryan C, Byrne S, Kennedy J, O'Mahony D. STOPP (Screening Tool of Older Person's Prescriptions) and START (Screening Tool to Alert doctors to Right Treatment). Consensus validation. Int J Clin Pharmacol Ther. 2008;46(2):72-83.
2. Aparasu RR, Mort JR. Inappropriate prescribing for the elderly: beers criteria-based review. Ann Pharmacother. 2000;34(3):338-46.
3. Mangin D, Bahat G, Golomb BA, Mallery LH, Moorhouse P, Onder G, et al. International Group for Reducing Inappropriate Medication Use & Polypharmacy (IGRIMUP): Position Statement and 10 Recommendations for Action. Drugs Aging. 2018;35(7):575-87.
4. Salahudeen MS, Hilmer SN, Nishtala PS. Comparison of anticholinergic risk scales and associations with adverse health outcomes in older people. J Am Geriatr Soc. 2015;63(1):85-90.
5. Boustani MA, Campbell C, Munger S, Maidment I, Fox C. Impact of anticholinergics on the aging brain: a review and practical application. Aging Health. 2008;4(3).
6. Lane R, Baldwin D. Selective serotonin reuptake inhibitor-induced serotonin syndrome: review. J Clin Psychopharmacol. 1997;17(3):208-21.
